# Sensitization to Disperse Blue Dye 124 in Triveneto Region from 1997 to 2021 and Its Potential Occupational Role

**DOI:** 10.3390/life15111711

**Published:** 2025-11-05

**Authors:** Nicholas Zampa, Serena Romanelli, Anna Belloni Fortina, Erika Giulioni, Luca Cegolon, Francesca Larese Filon

**Affiliations:** 1 Unit of Occupational Medicine, University of Trieste, 34127 Trieste, Italy; nicholas.zampa@studenti.units.it (N.Z.); serena.romanelli@studenti.units.it (S.R.); luca.cegolon@units.it (L.C.); 2 Department of Women’s and Children’s Health, University of Padua, 35122 Padova, Italy; anna.bellonifortina@unipd.it; 3 Divisione Dermatologica, Azienda Ospedaliera Santa Maria degli Angeli, 33170 Pordenone, Italy; erika.giulioni@asfo.sanita.fvg.it

**Keywords:** disperse blue dye 124, patch test, epidemiology, allergic contact dermatitis

## Abstract

**Objective**: To investigate the sensitization to the dispersal of blue 124, a synthetic dye used in textile applications. The chemical properties of this dye allow it to migrate from fabrics to the skin, posing a risk for sensitization and allergic reactions. **Materials and Methods**: A retrospective analysis of 30,629 consecutive patch test data from 1997 to 2021 in the Triveneto region (Italy) was performed using disperse blue 124 1% in petrolatum. Data were analyzed to assess trends in sensitization rates across different demographics and occupational groups. **Results**: The prevalence of sensitization to disperse blue 124 was 2.5% (n. 780 patients) and declined over the considered period, reaching a prevalence of approximately 1.5–1.9% in recent years. Sensitization was slightly higher in women (2.7%) compared to men (2.3%, *p* = 0.053), and in 36–65-year-old individuals (*p* < 0.05). Painters and textile workers presented a mild increase in sensitization, without reaching the statistical significance. **Discussion**: Disperse blue 124 sensitization declined significantly over the considered period, probably as result of the reduced use of this dye in textiles available on the Italian market. Its occupational role is limited. **Conclusions**: Contact dermatitis associated with disperse blue 124 declined over the considered period, but it is still above 1%, indicating the need for monitoring.

## 1. Introduction

Disperse dyes are used for the dyeing of synthetic textiles made from fibers composed exclusively of polyester, acetate, and nylon, or from a blend of these materials with other fiber types. However, they are not employed for dyeing natural fibers, such as wool, cotton, and linen [1,2,3]. Unlike other dyes, disperse dyes can leach onto the skin, especially from low-quality textiles [4]. This characteristic enhances their potential for causing allergic reactions. Indeed they are the most prevalent causes of textile-related allergic contact dermatitis [2,5].

Epidemiological studies have shown an increased prevalence of sensitization to disperse blue dyes among workers using uniforms treated with disperse blue [6,7,8,9,10], textile industries workers [11] and occupational groups exposed to cross-reacting dyes, such as hairdressers [12].

Currently, textile dye mixes containing disperse blue 35, disperse orange 1 and 3, disperse red 1 and 17, disperse yellow 3, all at 1.0%, and disperse blue 106 and disperse blue 124, both at 0.3%, are included in the European baseline series [13]. However, Nijman et al. [13] assert that it is beneficial to test individual textile dyes in addition to dye mix in patients suspected of having a textile dye allergy.

Disperse orange 3, disperse orange 1, disperse blue 124, and disperse blue 106 are the main culprits [2,14,15,16]. However, both disperse oranges cross react with *p*-phenylenediamine, while both disperse blues are the most important dye haptens. For that reason, it is important to verify the trend of disperse blue over time.

In the Triveneto patch test database, disperse blue 124 was tested regularly until 2021, after which it was replaced with textile dye mix.

### Objectives of the Study

This paper aimed to analyze data from patch test databases in the Triveneto region from 1997 to 2021 to elucidate the patterns of sensitization to disperse blue 124 dye. By examining demographic factors and occupational exposure risks, this study seeks to give valuable insights into effective prevention strategies and enhance awareness of textile-related allergies.

## 2. Materials and Methods

### 2.1. Study Design

This is a multicenter study that analyzed sensitization patterns to disperse blue 124 dye in the patch test database of the Triveneto region.

### 2.2. Participants

The Triveneto patch test database has been already described in several publications [17,18,19]. This study included 30,629 patients who underwent patch testing for suspected allergic contact dermatitis. Patch tests were performed in the departments of dermatology or occupational medicine of the NEICDG (North-East Italy Contact Dermatitis Group which involved Padua, Trieste, Pordenone, Belluno, Rovigo, and Trento-Bolzano, the latter being aggregated for analysis) [20]. Individual characteristics and personal and family history of atopic diseases (asthma and/or allergic rhino-conjunctivitis with at least one positive prick test to relevant aeroallergens) were collected from a standardized questionnaire. Subsequently, all patients underwent a clinical examination. All patients were categorized into occupational groups, consolidating similar job types into broader categories (e.g., the ‘Health Workers’ category). Those without formal employment were classified into three distinct groups: students, retirees, and the unemployed. Clerks were selected as the reference group, as their sensitization profiles are likely unaffected by job-related exposure. In addition to occupational categories, specific body areas (such as the fingers, palms, and back of the hands) were grouped into larger categories (e.g., ‘hands’). To minimize confounding factors, sex and age differences were considered, as outlined in the Section 2.4.

### 2.3. Patch Testing Procedure

Patch tests were conducted following standardized protocols. The procedure consisted in the use of Finn Chambers on Scanpor tape (Epitest, Tuusula, Finland) and haptens were purchased from FIRMA (Firenze, Italy) in order to perform patch tests with the European baseline series [21]. Disperse blue 124 dye (CAS number 61951-51-7) prepared in PET at 1% was tested for all the period considered. Allergens were applied to the skin on the upper back using aluminum patches, which were secured for 48 h to facilitate penetration. According to ICDRG guidelines [22], reactions were assessed at the moment of the removal (D2) and after 72 h (D3) or 96 h (D4) for delayed hypersensitivity responses. Patch tests resulting in +, ++, or +++ in the second examination (D3 or D4) were considered positive, while ± and ‘?’ were considered negative.

### 2.4. Statistical Analysis

Statistical analysis was performed using STATA™ v. 17.0 (Stata Corp., LP, College Station, TX, USA) to evaluate the significance of sensitization rates across different demographics and occupational categories. Descriptive statistics summarized patient characteristics, while inferential statistics assessed correlations between sensitization and various risk factors. Categorical data were organized into k × k contingency tables and analyzed using the chi-square test. The sensitization to disperse blue 124 dye was assessed through univariable and multivariate logistic regression analysis, with sex and age (treated as a continuous variable).s. Odds ratios (ORs) and 95% confidence intervals (CIs) were derived from the coefficients and standard errors obtained from the logistic regression output. A trend analysis for disperse blue 124 dye sensitization across ordered groups was conducted using Cuzick’s test for trend. Patients with missing data for relevant variables were excluded from the analysis. A *p*-value of <0.05 was established as the threshold for statistical significance.

### 2.5. Ethical Considerations

All patients underwent patch testing for diagnostic purposes.

The study adhered to ethical guidelines for research involving human subjects. Informed consent was obtained from all participants prior to their inclusion in the study, ensuring confidentiality and the right to withdraw at any time of the study. The Ethical Committee approved the study (n. 092/2018).

## 3. Results

A total of 30,629 patients, including 9935 males and 20,694 females, were investigated. The mean age was 44.1 ± 17.3 years for males and 43.6 ± 17.2 years for females (*p* = 0.02), with a notable prevalence of individuals over 40 years of age (53.5% for both sexes). Occupational dermatitis was diagnosed in 2520 participants, 998 males (10.1%) and 1522 females (7.4%). A positive history of atopic dermatitis was reported by the 9.6% of males (856 cases) and 10.3% of females (1912 cases). The most frequently affected anatomical sites were the hands (39.9% among males and 34.6% among females) and face (12.5% for males and 22.9% for females), with leg involvement being secondary.

Table 1 reports the characteristics of the population in relation to sensitization to disperse blue 124, which was found to be positive in the 2.5% of patients (n. 780 individuals).

Individuals sensitized to disperse blue 124 were older (*p* = 0.053) and mainly women with facial dermatitis. The prevalence of hand dermatitis resulted lower in patients sensitized to disperse blue 124 than in non-sensitized patients (*p* < 0.008).

Table 2 reports the characteristics of subjects sensitized to disperse blue 124 in women and men. Women resulted significantly older than men with more facial dermatitis (*p* = 0.008), while men presented more frequently hands dermatitis compared to women (*p* = 0.025).

Table 3 reports disperse blue 124 dye sensitization prevalence in different age groups, considering individuals under 26 years old as a reference. Patients aged 36–65 years had a significantly higher prevalence of sensitization to disperse blue 124 compared to younger and older patients. Moreover, the groups 46–55 and 56–65 years old had the higher prevalence of strong reactions (+++) compared to other age classes. Comparing the intensity of reactions between sexes, men presented a significantly lower risk to having a strong reaction (+++) at unvariable logistic regression (OR 0.29; 95% CI 0.17–0.47).

Table 4 reports the prevalence of sensitization to disperse blue 124 dye in occupational categories.

Textile workers (5.88%) and painters (3.92%) had the higher prevalence of sensitization. We analyzed cases that tested positive for disperse blue 124 (Table 5), of which only two cases were classified as occupational, due to contact with protective clothing.

Longitudinal analysis indicated a decreasing trend in sensitization prevalence over the study period (1996–2021) in both sexes (Table 6 and Table 7).

Table 8 provides data on significant concomitant sensitizations, revealing that many individuals sensitized to disperse blue 124 dye also exhibited sensitivities to other allergens, such as disperse yellow 3 dye (OR 16.1; 95% CI 12.0–21.7), quinoline sulfate 1% (OR 5.9; 95% CI 3.4–10.5) and many others.

## 4. Discussion

In the 30,629 patients tested, 780 (2.5%) resulted in being sensitized to disperse blue 124 with a declining trend from over 3.5% in the period 1996–2000 to below 2.0% in the period 2016–2021. This percentage of sensitization is similar to that reported in other EU studies: Heratizadeh et al. in 2017 [14] reported a sensitization rate of 2.3% in Germany during the period 2007–2014, while Nijman et al. in 2023 [13], analyzing individuals with suspected textile dermatitis between 2015 and 2021, found a higher prevalence (4.8%). Moreover, for both authors disperse blue 124 was the most important disperse allergen. Uter et al. [23] (1995–1999) found a sensitization prevalence of 4.3% to disperse blue 124 and/or 106. In the USA, the prevalence of sensitization was higher, reaching 8% during the period 2000–2011 [24]. In Australia, sensitization prevalence to disperse blue 124 was 11.2%, based on 2069 patients tested with a textile series before 2011 [25]. In Italy, a 2014 study found that disperse blue 124 was the principal allergen in textile allergy [26], although only individuals with textile dermatitis were considered. In a study by Uter at al. [27] in 2003, high variability in sensitization to disperse blue 106/124 1% was observed among 3041 patients tested across 31 centers, with a prevalence of sensitization ranging from 0 to 6.2%. The study also identified an increase in women and older patients.

A decreasing trend in sensitization to disperse blue 124 was previously reported in the USA by the Mayo Clinic, comparing patch test results from the period 2006–2010 to 2001–2005 [28].

However, sensitization to disperse blue 124, was lower than 1% (0.9% on 1854 patch tests in the period 2017–2021 and 0.7% on 5594 patch tests in the period 2015–2016), and it was tested in only a minority of cases [29].

No recent EU data are available, as the standard series has included only Textile dye mix since 2015.

The declining trend in sensitization is likely attributed to the improved quality of textiles and the restricted use of disperse dyes in coloring synthetic textiles. An older study from 2012 found the presence of disperse dyes in only 3 out of 121 textile samples available in the market across Europe, Asia, and the United States, concluding that the use of disperse dyes is very limited [30].

The observed mild sex disparity in sensitization rates may reflect higher contact with synthetic textiles in women. This finding is consistent with the existing literature [31] which suggests a higher incidence of allergic contact dermatitis to textiles among females.

Age-related trends in sensitization suggest that middle-aged individuals may be at a higher risk, likely due to increased exposure to synthetic textiles. In contrast, younger individuals may be less exposed, owing to the reduced use of such disperse dyes in Italian textiles, given their known sensitization potential.

In patients sensitized to disperse blue 124, the sites most commonly affected were the hands and face, with a higher prevalence of sensitization on the face (23.3%) compared to non-sensitized individuals (19.4%). An increased prevalence of facial dermatitis was previously reported by Nijman et al. [13], with 20.4% of cases, and by Ryberg et al. in 2006. [32] Additionally, in textile allergy, other sites, such as the axilla, legs, and trunk, are frequently affected due to contact with textiles and sweating that can increase the leaching of the dye [5].

In individuals sensitized to disperse blue 124, we did not observe an increased risk of occupational dermatitis (7.8% vs. 8.2% in individuals sensitized or non-sensitized to disperse blue 124, respectively) confirming a predominant extra-occupational role. In our study, an increased prevalence of sensitization was found among painters and textile workers, and two of the five cases were occupational, being related to contact with blue coveralls. The other cases were classified as non-occupational and sensitization to other haptens was observed. This finding aligns with the literature, where reports are primarily associated with contact with uniforms that release disperse blue [8,11,12,13,14], with only a very old study reporting cases in workers involved in the textile industry [11].

The analysis of concomitant associations permitted us to identify many haptens significantly related to disperse blue sensitization: as expected, these were disperse yellow, other coloring agents, such as paraphenyldiamine (with low association in accord with the literature [33]), and cross-reactive substances belonging to the para-group (diaminodiphenylmethane, benzocaine, isopropylparaphenylenediamine [34]).

For contact allergens with a higher prevalence of sensitization, high copositivity rates are expected (nickel, cobalt, chromium, and fragrance mix I) [33]. Moreover, metals are potentially cross-reactive, though this co-sensitization has to be interpreted as related to the high prevalence of metals’ sensitization in our area [35,36].

An association was found with the rubber accelerators mercaptobenzothiazole and mercaptobenzothiazole mix, but not with other rubber accelerators, such as carbamates and thiurams. No available data in the literature are present, to the best of our knowledge, on this topic, suggesting that there is also a multiple exposure condition for other haptens.

This study has some limitations. Although it is based on a large sample of individuals, the populations included patients who sought healthcare services for suspected allergic dermatitis, which may introduce selection bias into the results. Additionally, the multi-centric design of the study could have influenced data collection practices across different centers, despite all participants adhering to a standardized protocol.

To the best of our knowledge, this study represents the largest and longest investigation reporting data on sensitization to disperse blue 124 in Italy.

## 5. Conclusions

The prevalence of disperse blue 124 sensitization is declining in Italy, likely due to the use of less sensitizing dyes in textiles. Its role in occupational dermatitis appears to be limited to a few cases involving reactions to synthetic coveralls worn during work. Moreover, co-sensitizations with various other allergens are common, suggesting limited relevance for many disperse blue 124 reactions.

## Figures and Tables

**Table 1:** Demographic characteristics and MOAHLFA (Men, Occupational Dermatitis, Atopic dermatitis, Hands, Legs, and Face dermatitis, Age ≥ 40 years) index of patients sensitized (n. 780) and non-sensitized (n. 29,849) to the disperse blue 124. * Statistical significant differences.

	Disperse Blue +	Disperse Blue −	Total	*p*-Value
	**N. 780 (2.5)**	**29,849 (97.5)**	**30,629 (100)**	
Mean age (years ± SD)	44.8 ± 15.8	43.7 ± 12.3	43.8 ± 17.2	0.053
Males n. (%)	228 (29.2)	9707 (32.5)	9935 (32.4)	0.053
Occupational dermatitis n. (%)	61 (7.8)	2453 (8.2)	2520 (8.2)	0.669
Atopic dermatitis n. (%)	66 (9.4)	2702 (10.1)	2768 (10.1)	0.551
Hands n. (%)	216 (31.5)	9298 (36.4) *	9514 (36.3)	0.008
Legs n. (%)	51 (7.5)	2068 (8.1)	2119 (8.1)	0.525
Face n. (%)	160 (23.3) *	4955 (19.4)	5115 (19.5)	0.011
Age ≥ 40 n. (%)	446 (59.7) *	15,916 (53.3)	16,382 (53.5)	0.001

**Table 2:** Demographic characteristics and OAHLFA (Occupational Dermatitis, Atopic dermatitis, Hands, Legs, and Face dermatitis, Age ≥ 40 years) index of women and men sensitized (n. 780) to the disperse blue 124. * Statistical significant differences.

	Women	Men	Total	*p*-Value
N. (row %)	552 (70.8)	228 (29.2)	780 (100)	
Mean age (years ± SD)	45.4 ± 15.6	43.3 ± 16.0	44.8 ± 16.0	0.04
Occupational dermatitis n. (%)	41 (7.4)	20 (8.8)	61 (7.8)	0.525
Atopic dermatitis n. (%)	46 (9.3)	20 (9.6)	66 (9.4)	0.551
Hands n. (%)	141 (29.0)	75 (37.7) *	216 (31.5)	0.025
Legs n. (%)	35 (7.2)	16 (8.0)	51 (7.5)	0.699
Face n. (%)	127 (26.1) *	33 (16.6)	160 (23.3)	0.008
Age ≥ 40 n. (%)	341 (61.8)	125 (54.8)	446 (59.7)	0.072

**Table 3:** Disperse blue 124 sensitization prevalence in age groups and reaction severity. * Statistical significant differences.

Age Groups (Years)	Total	Sensitized	+	++	+++	%	OR	CI 95%
<26	5073	103	60	28	15	2.03	1	
26–35	6433	149	88	39	22	2.32	1.14	0.88–1.47
36–45	5787	164	98	42	24	2.83	1.40 *	1.09–1.80
46–55	5073	156	85	37	34 *	3.08	1.53 *	1.18–1.96
56–65	4208	124	60	29	35 *	2.95	1.46 *	1.12–1.90
>65	4055	84	47	20	17	2.07	1.02	0.76–1.36
Total	30,629	780	438	195	147	2.55		

**Table 4:** Prevalence of sensitization to disperse blue dye 124 in different occupational categories.

Occupational Categories	Total N°	Disperse Blue 124 Positive N.	%
Clerks	6692	175	2.62
Pensioners	4394	100	2.28
Other occupations without defined exposure	3847	97	2.52
Housewives	3564	114	3.20
Biological and healthcare workers	3087	79	2.56
Mechanics	1485	34	2.29
Housekeepers and restaurateurs	1297	32	2.47
Construction workers	1178	35	2.97
Students	830	6	0.72
Unemployed	661	24	3.63
Other craftsmen	568	20	3.50
Wood craftsmen	440	4	0.91
Domestic workers	411	10	2.43
Hairdressers, barbers, and beauticians	388	7	1.80
Teachers	364	7	1.92
Shops and services	353	8	2.27
Drivers	280	4	1.43
Farmers and fishermen	257	7	2.72
Chemical workers	229	8	3.49
Armed forces	159	4	2.52
Painters	98	4	3.92
Cashiers	26	0	0
Textile workers	17	1	5.88
Total	30,629	780	

**Table 5:** Characteristics of positive cases among painters and textile workers.

Case	Gender	Age	Dermatitis Localization	Atopy	Occupational Dermatitis	Disperse Blue 124	Other Sensitization
Painter	Female	40	Hands	No	No	+	Chromate, cobalt, kathon
Painter	Female	55	Hands Legs	No	Yes	+++	Perù balsam, lanolin, neomycin, perfume mix, disperse yellow 3 dye, thimerosal
Painter	Female	41	Face	No	No	+	Nickel, sesquilactone terpene
Painter	Male	56	Arm	No	Yes	++	-
Textile worker	Male	24	Diffuse	No	No	+	-

**Table 6:** Disperse blue 124 dye sensitization prevalence in men by year classes. Temporal trends in year classes were investigated using univariable logistic regression and are reported as odds ratios (ORs) and 95% confidence intervals (CIs) considering 1996–2000 as reference category. * Statistical significant differences.

Years	Total	Sensitized Males	%	OR	CI 95%
1996–2000	2951	104	3.52	Ref	
2001–2005	2420	53	2.20	0.61 *	0.44–0.85
2006–2010	1775	25	1.41	0.47 *	0.32–0.67
2011–2015	1439	26	1.81	0.39 *	0.25–0.60
2016–2021	1350	20	1.50	0.41 *	0.25–0.66
Total	9935	228	2.29		

**Table 7:** Disperse blue 124 dye sensitization prevalence in women by year classes. Temporal trends were investigated using univariable logistic regression and are reported as odds ratios (ORs) and 95% confidence intervals (CIs). * Statistical significant differences.

Years	Total	Sensitized Females	%	OR	95% CI
1996–2000	6170	232	3.76	Ref	
2001–2005	4901	151	3.20	0.81	0.66–1.00
2006–2010	3787	51	1.35	0.34 *	0.25–0.43
2011–2015	3187	58	1.82	0.47 *	0.35–0.63
2016–2021	2649	50	1.90	0.49 *	0.36–0.67
Total	20,694	552	2.67		

**Table 8:** Statistical significant concomitant sensitizations to disperse blue 124. Association was investigated using univariable logistic regression and is reported as odds ratios (ORs) and 95% confidence intervals (CIs). All haptens are in petrolatum.

Hapten	Positive		OR	CI
	N°	%		
Disperse yellow 3 dye 1%	66	8.62	16.1	12.0–21.7
Quinoline sulfate 1%	14	1.86	5.9	3.4–10.5
Mercaptobendothiazole (MBT) mix	17	2.18	3.6	2.2–6.0
Benzocaine 5%	20	2.62	3.3	2.1–5.3
Isopropylparaphenylenediamine (IPPD) 0.1%	17	2.18	2.9	1.7–4.7
Potassium dichromate 0.5%	122	15.64	2.7	2.2–3.3
Mercaptobendothiazole (MBT) 2%	12	1.54	2.7	1.5–4.9
Lanolin alcohol 30%	31	3.97	2.6	1.8–3.7
Perfumes mix 1	120	15.38	2.4	1.9–2.9
Colophonium 20%	30	3.85	2.35	1.6–3.4
Ethylenediamine dihydrochloride 1%	22	2.82	2.3	1.5–3.5
Phenolformaldehyde resin (PFR2) 1%	19	2.44	2.3	1.4–3.6
Neomicyn sulfate 20%	37	4.74	2.1	1.5–3.00
Cobalt chloride hexahydrate 1%	136	17.44	2.1	1.7–2.5
Paraphenyldiamine (PPD) 1%	54	6.92	2.01	1.5–2.7
Diaminodiphenylmethane 0.5%	36	4.62	1.98	1.4–2.8
Nickel sulfate hexahydrate 5%	244	31.28	1.3	1.1–1.5

## Data Availability

The data presented in this study are available on request from the corresponding author. The data are not publicly available due to privacy and ethical restrictions. Anonymized data may be made available by the corresponding author upon reasonable request.

## References

[1] Mobolaji-Lawal M., Nedorost S. (2015). The Role of Textiles in Dermatitis: An Update. Curr. Allergy Asthma Rep..

[2] Malinauskiene L., Bruze M., Ryberg K., Zimerson E., Isaksson M. (2013). Contact allergy from disperse dyes in textiles–a review. Contact Dermat..

[3] Hunger K. (2003). Industrial Dyes Chemistry Properties Applications.

[4] ETAD Project G1033 (1997). Extractability of Dyestuffs from Textiles over a Normal Life Time of Use.

[5] Ryberg K., Agner T., Andersen K.E., Bircher A., Diepgen T., Foti C., Giménez-Arnau A., Gonçalo M., Goossens A., Johansen J.D. (2014). Patch testing with a textile dye mix—A multicentre study. Contact Dermat..

[6] Ameur K., Youssef M., Belhadjali H., Soua Y., Korbi M., Henchi M.A., Zili J. (2019). Occupational acute generalized exanthematous pustulosis induced by disperse dyes in a textile. Contact Dermat..

[7] Khanna M. (2001). Occupational contact dermatitis to textile dyes in airline personnel. Am. J. Contact Dermat..

[8] Wilson H.T., Cronin E. (1971). Dermatitis from dyed uniform. Br. J. Dermatol..

[9] Van der Veen J.P., Neering H., De Haan P., Bruynzeel D.P. (1988). Pigmented purpuric clothing dermatitis due to Disperse blu 85. Contact Dermat..

[10] Mota F., Silva E., Varela P., Azenha A., Massa A. (2000). An outbreak of occupational textile dye dermatitis from Disperse blu 106. Contact Dermat..

[11] Soni B.P., Sherertz E.F. (1996). Contact dermatitis in the textile industry: A review of 72 patients. Am. J. Contact Dermat..

[12] Iorizzo M., Parente G., Vincenzi C., Pazzaglia M., Tosti A. (2002). Allergic contact dermatitis in hairdressers: Frequency and source of sensitisation. Eur. J. Dermatol. EJD.

[13] Nijman L., Rustemeyer T., Franken S.M., Ipenburg N.A. (2023). The prevalence and relevance of patch testing with textile dyes. Contact Dermat..

[14] Heratizadeh A., Geier J., Molin S., Werfel T. (2017). Contact sensitization in patients with suspected textile allergy. Data of the I nformation N etwork of D epartments of D ermatology (IVDK) 2007–2014. Contact Dermat..

[15] Ryberg K., Goossens A., Isaksson M., Gruvberger B., Zimerson E., Bruze M. (2011). Patch Testing with a Textile Dye Mix in a Baseline Series in Two Countries. Acta Derm. Venereol..

[16] Isaksson M., Ale I., Andersen K.E., Diepgen T., Goh C.-L., Goossens R A., Jerajani H., Maibach H.I., Sasseville D., Bruze M. (2015). Patch Testing to a Textile Dye Mix by the International Contact Dermatitis Research Group. Dermatitis.

[17] Piapan L., Mauro M., Martinuzzo C., Larese Filon F. (2020). Characteristics and incidence of contact dermatitis among hairdressers in north-eastern Italy. Contact Dermat..

[18] Mauro M., Fortina A.B., Corradin T., Marino A., Bovenzi M., Larese Filon F. (2018). Sensitization to, and allergic contact dermatitis caused by, colophonium in north-eastern Italy in 1996 to 2016 with a focus on occupational exposures. Contact Dermat..

[19] Santarossa M., Mauro M., Belloni Fortina A., Corradin M.T., Larese Filon F. (2020). Occupational contact dermatitis in Triveneto: Analysis of patch test data of the North Eastern Italian Database from 1996 to 2016. Contact Dermat..

[20] Piapan L., Belloni Fortina A., Giulioni E., Larese Filon F. (2024). Sensitization to ethylenediamine dihydrochloride in patients with contact dermatitis in northeastern Italy from 1996 to 2021. Contact Dermat..

[21] Johansen J.D., Aalto-Korte K., Agner T., Andersen K.E., Bircher A., Bruze M., Cannavó A., Giménez-Arnau A., Gonçalo M., Goossens A. (2015). European Society of Contact Dermatitis guideline for diagnostic patch testing—Recommendations on best practice. Contact Dermat..

[22] Wilkinson D.S., Fregert S., Magnusson B., Bandmann H.J., Calnan C.D., Cronin E., Hjorth N., Maibach H.J., Malalten K.E., Meneghini C.L. (1970). Terminology of contact dermatitis. Acta Derm. Venereol..

[23] Uter W., Geier J., Lessmann H., Hausen B.M. (2001). Contact allergy to Disperse blu 106 and Disperse blu 124 in German and Austrian patients, 1995 to 1999. Contact Dermat..

[24] Wentworth A.B., Richardson D.M., Davis M.D.P. (2012). Patch testing with textile allergens: The mayo clinic experience. Dermat. Contact Atopic Occup. Drug.

[25] Slodownik D., Williams J., Tate B., Tam M., Cahill J., Frowen K., Nixon R. (2011). Textile allergy-the Melbourne experience. Contact Dermat..

[26] Lisi P., Stingeni L., Cristaudo A., Foti C., Pigatto P., Gola M., Schena D., Corazza M., Bianchi L. (2014). Clinical and epidemiological features of textile contact dermatitis: An Italian multicentre study. Contact Dermat..

[27] Uter W., Geier J., Hausen B.M. (2003). Contact allergy to Disperse blu 106/124 mix in consecutive German, Austrian and Swiss patients. Contact Dermat..

[28] Wentworth A.B., Yiannias J.A., Keeling J.H., Hall M.R., Camilleri M.J., Drage L.A., Torgerson R.R., Fett D.D., Prakash A.V., Scalf L.A. (2014). Trends in patch-test results and allergen changes in the standard series: A Mayo Clinic 5-year retrospective review (January 1, 2006, to December 31, 2010). J. Am. Acad. Dermatol..

[29] Zawawi S., Yang Y.W., Cantwell H.M., Drage L.A., Youssef M.J., Yiannias J.A., Davis M.D.P., Hall M.R. (2023). Trends in Patch Testing with the Mayo Clinic Standard Series, 2017–2021. Dermat. Contact Atopic Occup. Drug.

[30] Malinauskiene L., Zimerson E., Bruze M., Ryberg K., Isaksson M. (2012). Are allergenic disperse dyes used for dyeing textiles?. Contact Dermat..

[31] Ryberg K., Goossens A., Isaksson M., Gruvberger B., Zimerson E., Nilsson F., Björk J., Hindsén M., Bruze M. (2009). Is contact allergy to disperse dyes and related substances associated with textile dermatitis?. Br. J. Dermatol..

[32] Ryberg K., Isaksson M., Gruvberger B., Hindsén M., Zimerson E., Bruze M. (2006). Contact allergy to textile dyes in southern Sweden. Contact Dermat..

[33] Yang Y.W., Yiannias J.A., Voss M.M., Hall M.R., Youssef M.J., Davis M.D.P., Voelker D.H., Klanderman M.C., Mangold A.R. (2023). Systematic Identification of Copositivity Groups in Standard Series Patch Testing Through Hierarchical Clustering. JAMA Dermatol..

[34] Picardo M., Cannistraci C., Cristaudo A., De Luca C., Santucci B. (1990). Study on cross-reactivity to the para group. Dermatologica.

[35] Rui F., Bovenzi M., Prodi A., Belloni Fortina A., Romano I., Corradin M.T., Larese Filon F. (2013). Nickel, chromium and cobalt sensitization in a patch test population in north-eastern Italy (1996–2010). Contact Dermat..

[36] Basso P., Mauro M., Miani A., Belloni Fortina A., Corradin M.T., Larese Filon F. (2020). Sensitization to nickel in the Triveneto region: Temporal trend after European Union regulations. Contact Dermat..

